# Adolescent and Young Adult ME/CFS After Confirmed or Probable COVID-19

**DOI:** 10.3389/fmed.2021.668944

**Published:** 2021-04-29

**Authors:** Lindsay S. Petracek, Stacy J. Suskauer, Rebecca F. Vickers, Neel R. Patel, Richard L. Violand, Renee L. Swope, Peter C. Rowe

**Affiliations:** ^1^Departments of Pediatrics, The Johns Hopkins University School of Medicine and the Kennedy Krieger Institute, Baltimore, MD, United States; ^2^Departments of Physical Medicine and Rehabilitation, The Johns Hopkins University School of Medicine and the Kennedy Krieger Institute, Baltimore, MD, United States; ^3^Arundel Pediatrics, Arnold, MD, United States; ^4^Central Florida Heart Care, Maitland, FL, United States; ^5^Rick Violand, PT, LLC, Ellicott City, MD, United States

**Keywords:** chronic fatigue syndrome, myalgic encephalomyelitis, dysautonomia, postural tachycardia syndrome, Hoffman sign, COVID-19, mast cell activation, neurodynamics

## Abstract

**Introduction:** Fatigue is a common acute symptom following SARS-CoV-2 infection (COVID-19). The presence of persistent fatigue and impaired daily physical and cognitive function has led to speculation that like SARS-CoV-1 infection, COVID-19 will be followed by myalgic encephalomyelitis/chronic fatigue syndrome (ME/CFS).

**Methods and Results:** We describe three adolescent and young adult patients who had confirmed or probable COVID-19 infections early on during the pandemic and were referred for evaluation to the Chronic Fatigue Clinic at the Johns Hopkins Children's Center. All patients reported orthostatic intolerance symptoms within the first 2 weeks of illness, and 10-min passive standing tests were consistent with postural tachycardia syndrome. After 6 months of illness, all three patients met criteria for ME/CFS. Clinical features of interest included strong histories of allergies in all three patients, two of whom had elevations in plasma histamine. Each demonstrated limitations in symptom-free range of motion of the limbs and spine and two presented with pathological Hoffman reflexes. These comorbid features have been reported in adolescents and young adults with ME/CFS.

**Conclusion:** ME/CFS can be triggered by COVID-19 in adolescents and young adults. Further work is needed to determine the pathogenesis of ME/CFS after COVID-19 and optimal methods of treating these patients. Our preliminary study calls attention to several comorbid features that deserve further attention as potential targets for intervention. These include neuromuscular limitations that could be treated with manual forms of therapy, orthostatic intolerance and POTS for which there are multiple medications and non-pharmacologic therapies, treatable allergic and mast cell phenomena, and neurologic abnormalities that may require specific treatment. Larger studies will need to ascertain the prevalence of these abnormalities.

## Introduction

The occurrence of chronic fatigue and other symptoms following infection with SARS-CoV-2 (COVID-19) has fueled speculation that the COVID-19 pandemic will trigger a wave of new cases of myalgic encephalomyelitis/chronic fatigue syndrome (ME/CFS) (1, 2). As currently defined, the diagnosis of ME/CFS requires a duration of at least 6 months together with a substantial impairment in previously tolerated activities usually accompanied by profound fatigue, post exertional malaise (PEM), unrefreshing sleep, and either cognitive impairment or orthostatic intolerance (3). The diagnosis of ME/CFS requires a careful history and examination, and it should be brought into question if these symptoms are not present for the majority of the time and with at least a moderate severity.

Orthostatic intolerance refers to a group of clinical conditions in which symptoms of fatigue, lightheadedness, difficulty concentrating, and others are aggravated by quiet upright posture and are ameliorated by recumbency (4). Among those with ME/CFS, orthostatic intolerance is found in 90% of adults and over 95% of adolescents (3, 5–7). There has been some uncertainty about whether orthostatic intolerance is an early and primary contributor to ME/CFS symptoms, or if it develops as a secondary phenomenon due to reduced activity or some other aspect of disease pathophysiology. This question can be difficult to resolve because the onset of ME/CFS can be insidious. Even when there has been an obvious post-infectious onset, the diagnosis of ME/CFS is made long after the acute illness, making the timing of symptom recall subject to bias.

We recently evaluated three adolescents and young adults in whom COVID-19 illness had been confirmed with direct testing or was highly suspected based on the timing of the onset of symptoms in association with peak local and regional pandemic cases, close exposure to confirmed cases, along with characteristic clinical features such as anosmia. In all three, ME/CFS symptoms were prominent from the outset, as were symptoms and signs of orthostatic intolerance, consistent with an early contribution of circulatory disturbances to the pathogenesis of symptoms, before the onset of physiological changes due to inactivity. The preliminary findings of this case series have the potential to inform the investigation and treatment of what has been termed “long COVID” (8) or post-COVID-19 ME/CFS.

## Participants and Methods

For this case series, eligible participants were individuals who had a confirmed or probable exposure to COVID-19 during the pandemic period and who had been referred to the Chronic Fatigue Clinic at the Johns Hopkins Children's Center after April of 2020. All individuals underwent a careful history and physical examination by a clinician with experience in the evaluation of ME/CFS. As part of routine procedure in the Chronic Fatigue Clinic, all individuals completed the unidimensional Wellness score which asks, “On a scale of 0–100 (with 0 being dying and 100 being the best a person can feel), how would you rate yourself on average over the last month?” This scale correlates well with longer questionnaires that measure health related quality of life (9).

Based on prior publications from our group and others about risk-factors for ME/CFS, the physical examination included an evaluation of joint hypermobility, screening maneuvers to identify limitations in symptom-free range of motion of the limbs and spine, a careful neurologic examination for evidence of myelopathy, orthostatic testing, and ascertainment of symptoms consistent with allergic inflammation and mast cell activation syndrome (10–13).

All patients had a general physical examination that included the nine-point Beighton score, a commonly used and reliable measure of joint hypermobility. Joint hypermobility was considered present if the Beighton score was four or higher. As part of the neurologic examination, all patients had an ascertainment of deep tendon reflexes and a Hoffman sign. The Hoffman sign was performed with the patient seated and with the head and neck in a neutral position. With the patient's distal interphalangeal joint of the middle finger supported by the examiner's index finger, the examiner's thumb made an abrupt downward flicking of the patient's distal phalanx. The Hoffman sign was considered positive if there was flexion of the patient's ipsilateral thumb or index finger (14). Patients were assessed for neurodynamic dysfunction and range of motion using the following maneuvers commonly used in physical therapy practice: seated slump testing, ankle dorsiflexion, passive straight leg raise, the upper limb neurodynamic test 1 (also known as the upper limb tension test with a median nerve bias), prone knee bend, and prone press-up. Methods for performing the examination maneuvers have been described in detail elsewhere (10, 11).

All patients were tested for orthostatic intolerance using a 10-min passive standing test (3, 15). Blood pressure and heart rate were recorded at 1-min intervals while the patient was supine for 5 min, and then again at 1-min intervals while the patient was standing upright and motionless for 10 min with the upper back resting against the wall and with the heels two to six inches away. At completion of standing, patients had repeat heart rate and blood pressure measurements for two further minutes in the supine position. Patients were asked when supine and at 1- to 2-min intervals while upright to report changes in symptoms on a 0–10 scale with 0 meaning absence of the symptom and 10 being the worst severity imaginable. The diagnosis of postural orthostatic tachycardia syndrome (POTS) for individuals 12–19 years required at least a 40 beat per minute (bpm) increase in heart rate between the lowest supine value and the peak while standing; for those 20 and older, a 30 bpm increase was required (16).

The Institutional Review Board of the Johns Hopkins Medical Institutes had waived informed consent for a retrospective study using data collected as part of routine care.

## Case Report (Patient 1)

A 19-year-old male resident of Florida with a past history of Gilbert syndrome and allergies developed a cough, sore throat, headache, and fatigue on June 17, 2020. These symptoms began 3 days after a household exposure to a visiting relative who shortly thereafter had a positive SARS-CoV-2 RNA nucleic acid quantification test. Despite sleeping 3–4 h more than usual per night, this adolescent felt exhausted and flu-like, and his SARS-CoV-2 RNA nucleic acid quantification test was positive on June 18. He experienced a loss of sense of smell which persisted for several months. Both parents became ill at the same time, with confirmation of COVID-19 via a nucleic acid quantification test in his father and subsequent positive COVID-19 antibody tests in both.

Prior to his COVID-19 diagnosis, this patient was a college student and track and cross-country athlete, running on average 60–70 miles per week. Two weeks after the onset of his symptoms, his attempts to resume running resulted in an increased cough, labored breathing, and lightheadedness. By July 1, there was some improvement in the fatigue, and he was able to run three miles, however the cough remained. Throughout the rest of July, he continued to experience a decreased tolerance for running and an increase in post exertional malaise, characterized by lightheadedness, an increase in fatigue, and coughing. He also developed chest pressure, intermittent chest pain, and a significant increase in his heart rate after basic tasks such as walking to another room or showering. During very light activity (a game of cornhole, similar to bean bag toss) 2 months after the onset, his heart rate was 160–170 bpm for 20–30 min, followed by 3 days of PEM. A cardiac evaluation in mid-July, including an electrocardiogram (ECG), chest x-ray, and echocardiogram, revealed no abnormalities. A cardiopulmonary exercise test at 4 months after onset of illness showed a normal exercise ECG, but below average peak VO2 of 84% of predicted. A cardiac MRI showed no abnormalities. The troponin level was 0.009 mg/mL 6 weeks after the onset of illness.

We evaluated him 2 months after the onset of the COVID-19 infection, at which time his symptoms included constant fatigue, unrefreshing sleep, PEM after mild increases in activity, bi-frontal and bi-temporal headaches, chest pain, occasional cough, leg pain, insomnia, frequent awakening, and mild anxiety and depression. He also had a pre-COVID-19 history of mild asthma, allergic inflammation, and several food intolerances.

**Figure 1:**
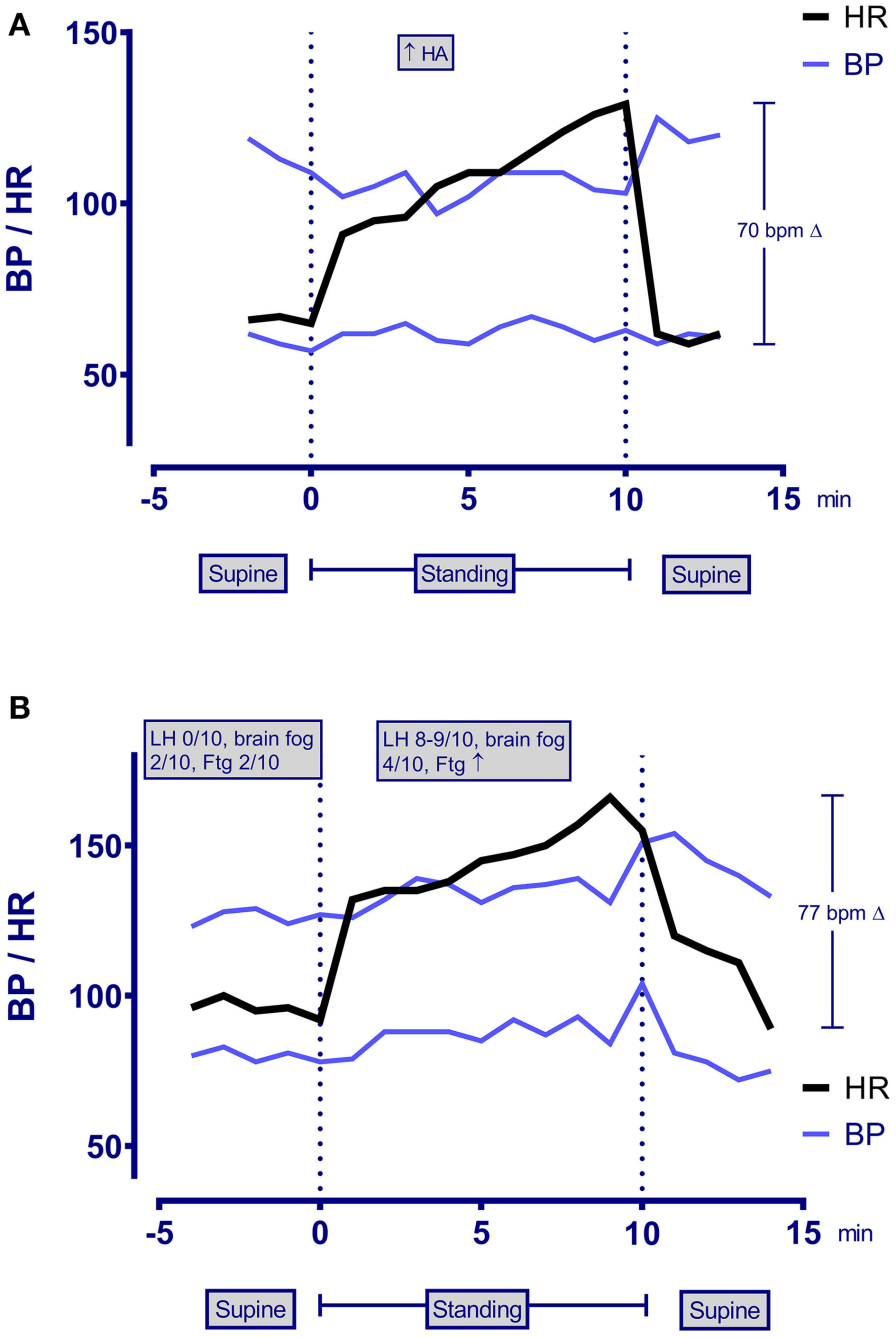
Heart rate, blood pressure, and symptom changes during a 10-min passive standing test in patient 1 **(A)** and patient 2 **(B)**. LH, lightheadedness; Ftg, fatigue; bpm, beats per minute.

His physical examination showed a healthy-looking young man in no distress. He had a Beighton score of 3/9 for >10 degrees of hyperextensibility of the right elbow and both knees. His neurologic examination showed 2+ symmetrical deep-tendon reflexes with a bilaterally positive Hoffman sign. He had limited symptom-free range of motion on a seated slump test, lacking 30 degrees of full leg extension of the right and 60 degrees of the left. Passive straight leg raise end-range was limited at 35 degrees bilaterally. Upper limb neurodynamic test 1 lacked 50 degrees of elbow extension on the left and 40 degrees on the right. To investigate the lightheadedness and fatigue, a 10-min passive standing test showed a 70 bpm difference between his lowest supine and peak standing heart rate, consistent with a diagnosis of postural orthostatic tachycardia syndrome (Figure 1, Table 2).

A month after onset, his complete blood count showed a WBC of 5.1, hemoglobin 16.1, and platelet count of 255. His comprehensive metabolic panel was normal with the exception of a total bilirubin of 1.5 (reference range, 0.2–1.1 mg/dL) consistent with his prior diagnosis of Gilbert syndrome. His CK was normal at 79 (reference range, 44–196 U/L). The sedimentation rate was 6. In light of his allergic history, testing was also performed to evaluate the presence of mast cell activation disorder. Serum tryptase was normal at 5.4 (reference range, <11.0 mcg/L) but chromogranin A was mildly elevated at 144 (reference range, 25–140 mg/mL), and his plasma histamine was elevated to 4.2 (reference range, ≤ 1.8 mg/mL); plasma histamine remained elevated on repeat testing 3 and 7 months after the onset of the illness.

After 6 months of illness, he satisfied the Institute of Medicine criteria for ME/CFS. Seven months post-COVID-19, his ability to function as he had pre-illness remains markedly decreased. His main symptoms consist of persistent fatigue, limited tolerance of exercise, unrefreshing sleep, PEM, lightheadedness, and headaches. He now tolerates two to three 15-min walks daily without provoking excessive tachycardia or PEM. Current treatments include Lexapro 5 mg daily for post-illness anxiety, loratadine 10 mg daily and famotidine 40 mg twice daily for the allergic symptoms and elevations in histamine, methylphenidate 10 mg each morning as a vasoconstrictor, along with compression garments and an increased sodium intake for POTS.

## Results

### Participants

In addition to the patient reported above, two other individuals were referred to the clinic between August and October 2020. The demographic and clinical data on the three participants are displayed in Table 1. As discussed above, patient 1 had a confirmed COVID-19 infection. Patients 2 and 3 had close contact with an individual with confirmed COVID-19 infection and developed anosmia and dysgeusia lasting up to 5 months. Nucleic acid quantification testing for COVID-19 was performed relatively late, 1 month after onset of illness in patient 2. Nucleic acid quantification testing for patient 3 was not performed due to lack of availability, but two further household members developed similar symptoms within days of exposure to her. Of interest, antibody testing later was negative for patients 2 and 3.

**Table 1:** Onset, diagnostic testing, and clinical features of confirmed and probable COVID-19 infection in the study participants.

**Patient**	**Age (yrs)**	**Sex**	**Onset of COVID-19 symptoms**	**COVID-19 PCR**		**COVID-19 antibody**		**Exposure**	**Distinctive COVID-19 symptoms**
				**Result**	**Date**	**Result**	**Date**		
1	19	M	6/17/2020	+	6/18/20	+IgG	1/19/21	Exposed to relative 3 days before onset. Other household contacts also developed COVID-19 symptoms and had positive PCR or antibody tests	Cough, sore throat, headache and fatigue at onset without fever followed by loss of sense of smell.
				+	7/01/20	+IgM	1/19/21		
				–	7/15/20				
2	30	F	3/19/2020	–	4/09/20	–	4/09/20	Exposed 2 days earlier to co-worker in an office two doors down the hall whose COVID-19 PCR test was positive	Mild cough, fatigue, and low-grade temperature elevation at onset followed by loss of taste and smell at day 5 (persisting for 5–6 weeks)
3	22	F	4/07/2020	Not performed		–	9/20/20	Exposed to a positive case and within days developed symptoms. In the next week, mother and boyfriend developed fever, myalgias, chills, headache, and shortness of breath	Low grade fever, sore throat, fatigue, and shortness of breath for 2 weeks.

*PCR, polymerase chain reaction/nucleic acid quantification test*.

### Criteria for ME/CFS

As shown in Table 2, all three participants met criteria for ME/CFS 6 months after the onset of symptoms. ME/CFS symptoms had been present from day one of the illness. None of the participants had a prior medical history of ME/CFS. Symptoms of orthostatic intolerance were also present at an early point of the initial respiratory illness, from 1 day to 2 weeks. All had profound POTS on testing several months after the onset, as shown in Table 2, with the increment between lowest supine and peak standing heart rate within the first 10 min well-exceeding the diagnostic criteria, together with reproduction of orthostatic symptoms.

**Table 2:** Responses to the 10-min passive standing test and features of ME/CFS in the study participants.

**Patient**	**Age**	**Orthostatic intolerance**							ME/CFS^*^						
		**Lowest supine HR**	**Peak HR standing**	**Δ** **HR**	**Supine BP**	**BP at peak HR**	**Symptoms during passive standing test**	**Onset of orthostatic symptoms during COVID-19**	**1. Substantial impairment in previously tolerated activities** **+** **fatigue**	**2. PEM**	**3. Unrefreshing sleep**	**4a. Cognitive impairment**	**4b. OI**	**Onset of fatigue**	**Wellness score at initial visit (0–100)**
1	19	59	129	70	109/57	103/63	Headache	Week 1	x	x	x	x	x	Day 1	40
2	30	89	166	77	127/78	139/93	Fatigue Blurry vision Brain fog Lightheadedness Increased Respiratory rate	Day 2	x	x	x	x	x	Day 1	20
3	22	75	129	54	94/61	NA	Lightheadedness Fatigue	By week 2	x	x	x		x	Day 1	42

*HR, heart rate; BP, blood pressure; PEM, post-exertional malaise; OI, orthostatic intolerance; ME/CFS, myalgic encephalomyelitis/chronic fatigue syndrome; Δ, change in heart rate between lowest supine and peak heart rate standing; NA, not available*.

* *The diagnosis of ME/CFS was made after a duration of 6 months of symptoms*.

### Other Clinical and Allergic Phenomena

Allergic symptoms and history were present in all participants as displayed in Table 3. Notably, one patient had a history of oral allergy syndrome, another had urticaria after exposure to citrus, and the third had cutaneous features consistent with mast cell activation (dermatographism and facial flushing). Table 3 also displays the pertinent physical examination findings. Two of the three patients had an abnormal Hoffman sign. Patient 2 met criteria for hypermobile Ehlers Danlos syndrome. Neurodynamic testing measures commonly used in physical therapy to assess for changes in, among other things, neural gliding function in the trunk and limbs, revealed restricted range of motion findings in all three patients.

**Table 3:** Past medical history and physical examination abnormalities.

**Patient**	**Prior medical history**	**Allergic phenomena**	**Examination abnormalities**		
			**Beighton score (0–9)**	**Hoffman sign**	**Comments**
1	Gilbert Syndrome	Allergies to pollens, grasses Immunotherapy for 3 yrs (ages 10–13) Oral allergy syndrome to carrots, cashews, and cherries Mild asthma Family history positive for allergic rhinitis and oral allergy syndrome Post COVID-19 plasma histamine 3.5 (normal ≤ 1.8 mg/ml)	3	+ bilateral	**Physical maneuvers:** Seated slump testing elicited stretch at 150° leg extension on the right, 120° on the left Passive straight leg raise elicited stretch at 30° bilaterally with end range 35° Upper limb neurodynamic test 1 elicited stretch and guarding at 130° of elbow extension on the left, 140° on the right Mild thoracic hypomobility on prone press-up
2	Presyncope (2× 10 years earlier) Migraine Post-pneumonia exhaustion (9 yrs earlier) Anxiety Discrete episode of depression (10 yrs earlier) with no intervening symptoms Dysmenorrhea	Allergic rhinitis (cat, dust, pollens) Urticaria after exposure to citrus in childhood Eczema Mild asthma Post COVID-19 plasma histamine 4.2 and 3.4 (normal ≤ 1.8mg/ml)	5	+ bilateral	**Neurological:** Brisk 3+ deep tendon reflexes at triceps and patella Fine resting tremor in hands **Physical maneuvers:** Upper limb neurodynamic test 1 elicited stretch at 160° of elbow extension on the left, 170° on the right Mild pectoralis minor tenderness L > R
3	Celiac disease Anorexia Dysmenorrhea	Facial flushing with activity Dermatographism Family history positive for histamine sensitivity	3	–	**Cutaneous:** Diffuse skin erythema Acrocyanosis in lower limbs **Physical maneuvers:** Mild pectoralis minor tenderness

## Discussion

This case series describes one patient with confirmed SARS-CoV-2 infection and two with highly probable SARS-CoV-2 infection in whom the diagnostic criteria for ME/CFS were satisfied by month 6 of the illness. Fatigue has previously been reported as a common acute symptom of COVID-19, with a prevalence reaching 80% in the first month and remaining as high as 53% 2 months after the onset (17). Our work parallels the findings of a recent survey of 3,762 international COVID-19 patients by Davis and colleagues (8). They reported fatigue, PEM, and cognitive dysfunction as the three most common symptoms persisting after 6 months. Of the total survey population, 2,308 respondents reported tachycardia. Of all the respondents, only 8.43% were hospitalized. The probability of having fatigue was high from day one and the probability of having PEM peaked and plateaued around week six.

Several observations from our case series warrant further emphasis. First, all three individuals had relatively mild respiratory symptoms and none required hospitalization. This is consistent with observations from other groups that persistent fatigue following COVID-19 infection is independent of the severity of the initial infection (18). The potential for marked impairment in function after relatively mild respiratory illnesses contrasts with the emergence of ME/CFS after other infectious illnesses. In the 10–13% of individuals who meet criteria for ME/CFS 6 months following infectious mononucleosis, the risk of ME/CFS is related to the severity of the initial infection (19–21). The occurrence of ME/CFS after relatively mild viral illnesses raises the question of how many ME/CFS cases before the COVID-19 pandemic might have been due to mild, sub-clinical, or asymptomatic infections.

Second, as early as within the first 2 days and certainly within the first 2 weeks, all three developed symptoms of orthostatic intolerance and ultimately met criteria for POTS, which has been reported as a post-COVID-19 phenomenon by several groups (8, 22–25). Similar reports of dysautonomia followed the SARS-CoV-1 pandemic (26). While the 10-min passive standing tests to confirm the diagnosis of POTS were conducted 5–7 months after the onset of COVID-19, the orthostatic symptoms were present at an early point, suggesting that autonomic symptoms could have been a direct result of the viral infection rather than a consequence of inactivity or deconditioning. In those diagnosed with POTS following COVID-19 infection, there has been a variable onset of orthostatic symptoms. Miglis (22) reported palpitations developing on day two of the COVID-19 illness in a 26-year-old female nurse. She had noticed tachycardia by day seven of the illness. Kanjwal et al. reported a 36-year-old female who began to develop fatigue, dizziness, and palpitations with postural changes 3–4 weeks after the initial COVID-19 infection (25). Given the delay between the acute illness and the orthostatic symptoms, these authors speculated that autoimmunity was a likely mechanism for the onset of POTS, as has been reported by several groups (27–30). Novak (23) described a similar patient who developed orthostatic symptoms 1 month after the onset of acute COVID-19 infection, in whom intravenous immunoglobulin was partially successful. Further attention to the timing of the onset of orthostatic symptoms has the potential to help determine whether autonomic symptoms result from direct infection by the virus, sympathetic activation as part of the immune response, mast cell activation, or autoimmunity (31). In heterogeneous disorders like POTS, each of these mechanisms could be important for subsets of patients and might warrant different approaches to treatment. Based on these reports of orthostatic intolerance in patients with chronic symptoms post-COVID-19, and the high prevalence of orthostatic intolerance in those with established ME/CFS, we would recommend at least 10 min of orthostatic testing for all patients reporting chronic fatigue in the context of long COVID.

Third, range of motion impairments have been found more commonly in individuals with ME/CFS than in healthy controls and are important in the pathogenesis of symptoms (11, 32). The application of a longitudinal neural strain such as that imposed by a straight leg raise maneuver is capable of aggravating fatigue and other symptoms for at least 24 h (33). The mechanism for these range of motion impairments is unknown, but possible explanations include the result of previous musculoskeletal injuries, excessive guarding around hypermobile joints, post-infectious inflammation, and reduced activity in response to an illness (34). While two of our three patients presented with range of motion impairments, their pre-COVID-19 range of motion measurements are unknown. Therefore, it is impossible to tell whether these impairments preceded the onset of ME/CFS or were a result of the viral illness.

Fourth, all patients in our series had prominent histories of allergic inflammation. Afrin and colleagues and others (35–37) have hypothesized that mast cell activation can play an important pathophysiologic role in the hyperinflammatory response to COVID-19. Two of the three patients have had sustained elevations in plasma histamine, and both have had improvement in fatigue and cognitive dysfunction in response to treatment with drugs that block the H1 histamine receptor, consistent with mast cell activation. Further work is needed to determine the prevalence of similar allergic histories and evidence of mast cell activation syndrome (38) in others with prolonged symptoms for ME/CFS after COVID-19. If allergic inflammation and mast cell activation are common, it would be important to determine whether medications with antihistamine properties or medications that are capable of stabilizing mast cell membranes will prove effective in ameliorating the symptoms of post-COVID-19 ME/CFS.

Fifth, neuroanatomic abnormalities have been recognized in a subset of those with ME/CFS symptoms, including Chiari malformation, congenital or acquired cervical stenosis, and instability at the skull base or in the spine. Heffez (39) has described 270 patients with fibromyalgia among whom 64% had hyper-reflexia and 26% had a positive Hoffman sign (39). Our research group has reported a series of three patients in whom congenital or acquired cervical stenosis was a treatable cause of refractory orthostatic intolerance and other ME/CFS symptoms (12). The presence of abnormal Hoffman signs in two of our post-COVID-19 patients was unexpected after an acute illness. Long term study will be needed to determine whether these abnormalities were transient and related to the viral infection or persistent and related to underlying neuroanatomic abnormalities that predisposed patients to prolonged symptoms and autonomic dysfunction.

## Limitations

This report of three cases establishes the potential for adolescents and young adults who have mild respiratory illnesses from confirmed or probable COVID-19 to develop prolonged symptoms consistent with ME/CFS. While one patient had proven COVID-19 infection, we could not confirm the presence of COVID-19 in the other two, a problem that was complicated by the lack of availability of testing early in the pandemic. These two patients had highly probable COVID-19 based on their close temporal exposure to confirmed cases and based on characteristic symptoms such as anosmia and dysgeusia. A curious finding was that none of the two probable cases developed antibodies to COVID-19 in the convalescent phase of their illness. Whether impaired production of antibodies is a risk-factor for prolonged symptoms after COVID-19 or is related to a sampling anomaly will need to be assessed in larger samples. Because these patients were referred to a specialist clinic at a tertiary care center, we cannot know whether the clinical features we observed will prove to be common across the general population of those with prolonged symptoms after COVID-19.

## Conclusion

Our evaluation of this sample of three patients suggests that ME/CFS can be triggered by confirmed or probable COVID-19 in adolescents and young adults. Komaroff and Bateman (1) predict that over 10 million new cases of ME/CFS will be triggered by COVID-19 globally. Further work is needed to determine the pathogenesis of ME/CFS after COVID-19 and how to treat these patients optimally to promote their return to their pre-COVID-19 quality of life. Our study identifies several comorbid features that could be treated including ROM limitations that could respond to manual forms of therapy, orthostatic intolerance and POTS for which there are multiple non-pharmacologic and pharmacologic therapies, treatable allergic phenomena, and neurologic abnormalities that may require specific treatment. If non-pandemic ME/CFS provides any guidance, the treatments for each patient are likely to vary based on the contributions of comorbid conditions. Whether post-COVID-19 patients are more homogeneous remains to be determined, but the expected number of new cases provides an opportunity to study diagnostic procedures, including standing and head-up tilt testing, as well as candidate therapies in an organized manner. These efforts also have the potential to inform the treatment of individuals with non-pandemic ME/CFS.

## Data Availability Statement

The datasets presented in this article are not readily available because: the main data from this case series is included in this article. Requests to access the datasets should be directed to prowe@jhmi.edu.

## Ethics Statement

The studies involving human participants were reviewed and approved by Johns Hopkins Institutional Review Board. Written informed consent for participation was not required for this study in accordance with the national legislation and the institutional requirements. Written informed consent was obtained from the individual(s) for the publication of any potentially identifiable images or data included in this article.

## Author Contributions

LP wrote the initial draft and was involved in critical review of the final manuscript. SS, RLV, NP, RS, and PR were instrumental to the clinical care of patients, and have contributed to the writing and review of the manuscript. RLV contributed to the writing and critical review of the manuscript. All authors contributed to the article and approved the submitted version.

## Conflict of Interest

RLV was employed by company Rick Violand, PT, LLC. The remaining authors declare that the research was conducted in the absence of any commercial or financial relationships that could be construed as a potential conflict of interest.
